# Retinoblastoma in pregnancy

**DOI:** 10.4103/0301-4738.71686

**Published:** 2010

**Authors:** Charudatt Chalisgaonkar, M K Rathore, P C Dwivedi, Pankaj Choudhary, Sujata Lakhtakia, Kriti Sharma

**Affiliations:** Department of Ophthalmology, S. S. Medical College, Rewa (M. P.), India

Dear Editor,

We read the article titled, ’Presentation of Retinoblastoma in Pregnancy’ by Nandedkar *et al*. with interest and congratulate them for reporting of such a rare case.[1] However, we would like to make a few points about the same.

The authors have mentioned in their article that calcification has never been reported in cases of adult retinoblastoma. However, on reviewing literature we found that calcification, although rare, has been mentioned in previous case reports.[2,3] Jyotirmay Biswas *et al*, have reported a spec of calcification on computed tomography in one of the three cases of adult retinoblastoma reported by them.[2]

The authors have mentioned the absence of retinocytoma in their case; however, the presence of pre-existing retinoma or retinocytoma cannot be excluded, because there is no mention of previous records of normal fundus in this case.

The association of pregnancy with retinoblastoma appears to be speculative, and it may be coincidental until proved otherwise. An exhaustive search of published literature did not reveal evidence of increased incidence of malignancies in pregnancy. The immunosupression and excessive circulating mutagens in pregnancy, as suggested by the authors, will effect only genetically predisposed persons. There are rare reports of the association of pregnancy with ocular tumors such as uveal melanoma and hemangioma, however, on estrogen and progesterone hormone receptor analysis, such associations were found to be coincidental.[4] Similar studies in cases of adult retinoblastoma in future may establish its possible association with pregnancy.
